# Animal Models of Varicella Zoster Virus Infection

**DOI:** 10.3390/pathogens2020364

**Published:** 2013-05-13

**Authors:** Kristen Haberthur, Ilhem Messaoudi

**Affiliations:** 1 Department of Microbiology and Molecular Immunology, Oregon Health and Science University, Portland, OR 97239, USA; E-Mail: haberthu@ohsu.edu; 2 Division of Biomedical Sciences, University of California Riverside, Riverside, CA, 92521, USA

**Keywords:** varicella zoster virus, guinea pigs, SCID-humanized mouse model, non-human primates, simian varicella virus

## Abstract

Primary infection with varicella zoster virus (VZV) results in varicella (chickenpox) followed by the establishment of latency in sensory ganglia. Declining T cell immunity due to aging or immune suppressive treatments can lead to VZV reactivation and the development of herpes zoster (HZ, shingles). HZ is often associated with significant morbidity and occasionally mortality in elderly and immune compromised patients. There are currently two FDA-approved vaccines for the prevention of VZV: Varivax^®^ (for varicella) and Zostavax^®^ (for HZ). Both vaccines contain the live-attenuated Oka strain of VZV. Although highly immunogenic, a two-dose regimen is required to achieve a 99% seroconversion rate. Zostavax vaccination reduces the incidence of HZ by 51% within a 3-year period, but a significant reduction in vaccine-induced immunity is observed within the first year after vaccination. Developing more efficacious vaccines and therapeutics requires a better understanding of the host response to VZV. These studies have been hampered by the scarcity of animal models that recapitulate all aspects of VZV infections in humans. In this review, we describe different animal models of VZV infection as well as an alternative animal model that leverages the infection of Old World macaques with the highly related simian varicella virus (SVV) and discuss their contributions to our understanding of pathogenesis and immunity during VZV infection.

## 1. Introduction

### 1.1. Virological Features of VZV

Varicella zoster virus (VZV) is a neurotropic alphaherpesvirus of the *Varicellovirus* genus, and the causative agent of varicella (chickenpox) and herpes zoster (HZ, shingles). Like other members of the alphaherpesvirus subfamily, VZV establishes a latent infection in sensory ganglia [1]. The structure of VZV is also indistinguishable from other herpesviruses and is comprised of four main elements: the core, the nucleocapsid, the tegument, and the envelope [1]. The core has been described as a loose fibrillar cage of strands that surround a dense cylindrical core of DNA fibers and is encompassed by the nucleocapsid [2]. The unstructured proteinaceous layer between the nucleocapsid and envelope is called the tegument and contains an assortment of viral proteins believed to modulate the host environment to meet the needs of the virus [3]. The envelope is derived from patches of altered cellular membranes and contains numerous glycoprotein spikes [4]. The VZV genome is the smallest of the human herpesviruses at approximately 125kb and is organized into a unique long region (UL) flanked by terminal long (TRL) and internal long (IRL) repeats, and a unique short region (US) flanked by internal short (IRS) and terminal short (TRS) repeats [5]. VZV encodes 70 unique open reading frames (ORFs) [6] and viral gene transcription during lytic infection appears to be coordinated and follows a temporal program with immediate-early, early, and late genes [7]. 

### 1.2. VZV Transmission and Clinical Manifestations

There is strong clinical evidence that primary VZV infection occurs through the inhalation of virus-laden respiratory droplets [8,9] or airborne virions from varicella lesions [10], or by contact with infectious vesicular fluid [11]. The incubation period of varicella can range from 10 to 21 days [12]. Initially, it was believed that VZV replication followed a pattern of dual viremia [11]. However, recent studies indicate that VZV is more likely to follow a single viremia model. Studies propose that VZV infects dendritic cells (DCs) within the mucosal epithelia, which then traffic to the regional lymph nodes or tonsils where they transfer VZV to T cells [13,14]. The infected T cells then home to the skin to infect dermal fibroblasts and keratinocytes, resulting in characteristic varicella [15,16]. Given that VZV pneumonia is one of the main complications of VZV infection in adults [17,18], another possibility is that VZV also replicates in the lungs after initial inhalation. It has been postulated that tonsillar T cells can be directly infected following inhalation of viral particles and transport VZV to the skin based on *in vitro* infectivity data [16,19,20] as well as the ability of intravenously transferred tonsillar T cells infected in vitro to result in skin lesions in the humanized SCID mouse model [20] (discussed in greater detail below). Varicella is characterized by the appearance of a vesicular rash, fever, headache, and loss of appetite [12]. In the absence of *ex vivo* analysis during VZV infection, the exact mode of VZV trafficking remains unknown. 

It is unclear how or when VZV travels to the ganglia following primary infection, but two hypotheses exist. The first proposes that the sensory nerves of the dorsal root terminating within the dermis become infected either by the release of cell-free virus following epidermal infection or due to the cell-to-cell fusion of infected cells with neuronal axons [21,22]. The second hypothesis postulates that VZV accesses distal neurons via a hematogenous route, specifically by infected T cells during the viremic stage [21]. VZV would then enter neuronal cell body by an unknown mechanism to establish a latent infection [23].

VZV reactivation typically presents as herpes zoster (HZ), although it can be asymptomatic at times as has been documented in astronauts during and shortly after spaceflight [24,25], and as suggested by spikes in VZV-specific antibody titers in the absence of clinical disease [26]. Approximately one million cases of HZ are reported annually in the US, and 60% of these cases occur in persons 50 years of age or older [27]. HZ can affect up to 30% of individuals during their lifetime [28], and 50% of persons 85 years will have had at least one episode of HZ (CDC: http://www.cdc.gov/shingles/hcp/clinical-overview.html). HZ begins with a prodromal phase characterized by pain, itching, or burning sensations in one to three dermatomes [23]. In most cases, this is followed by the development of a unilateral maculopapular rash on the affected area a few days later, which evolves into vesicles [28] that usually enter into the crusting phase within 10 days [1]. Although it is rarely life-threatening, HZ can result in significant morbidity [29]. The most common complication of HZ is post-herpetic neuralgia, which is debilitating pain that can persist for months to years after the resolution of the HZ rash [30]. Other complications include chronic ocular inflammation and permanent blindness in severe cases [31], vertigo and hearing loss [32], as well as myelitis and focal vasculopathies [33]. Asymptomatic reactivations can also cause neuropathic pain in the absence of rash, which is referred to as zoster sine herpete [34,35,36].

### 1.3. VZV Vaccines

There are currently two FDA-approved VZV vaccines, both of which contain the live-attenuated Oka strain of VZV: Varivax, for the prevention of varicella, and Zostavax, for the prevention of HZ. While Varivax contains on average 1350 plaque-forming units (PFU) per 0.5 mL of vaccine, Zostavax has a minimal potency of 19,400 PFU [37]. Varivax was licensed in the US for use in children 2–12 years old. Although the one-dose Varivax administration program resulted in ~80% reduction of varicella incidence, reports of breakthrough varicella in vaccinated children [38,39,40] prompted the CDC to implement a two-dose administration strategy in 2006. Since then, the incidence of chickenpox and annual varicella-related hospitalizations and deaths in the US has dramatically decreased by 97% [41]. Vaccination with Zostavax reduces the incidence of HZ by 51% and the burden of disease (including post-herpetic neuralgia) by 61% [37,42]. Unfortunately, immunological analysis following Zostavax vaccination in an elderly population showed that VZV-specific T cells and IgG titers declined significantly within the first year and returned to almost pre-vaccination levels three years later [43]. Furthermore, reports indicate that the vaccine efficacy is uncertain beyond five years post-vaccination [44].

## 2. Animal Models of VZV Infection

### 2.1. Guinea Pigs

Successful infection of guinea pigs with VZV only occurs with the use of a guinea pig-adapted strain [45], which is prepared by passaging the virus 10–30 times in fetal guinea pig cells [46,47,48,49] and in some cases is further propagated in human diploid cells [50]. Intranasal and subcutaneous inoculation of guinea pigs with the adapted virus results in seroconversion in both outbred Hartley and weanling guinea pigs [49,50], but the development of rash has been somewhat inconsistent. For instance, in one study, intramuscular VZV inoculation of inbred weanling guinea pigs resulted in the development of exanthema in 24/30 infected animals 3–7 days post-infection [46]. However, in a different study, intramuscular VZV inoculation of inbred weanling guinea pigs did not result in clinical disease [47]. Similarly, subcutaneous VZV inoculation of Hartley guinea pigs did not result in exanthema [50]. Evidence of VZV replication has also been inconsistent in this model. For instance, one study detected infectious VZV in nasopharyngeal sheddings of weanling guinea pigs after intranasal or environmental exposure to the virus, but viremia was rarely detected [49]. On the other hand, VZV DNA, but not infectious virus, was detected in PBMC from inbred weanling strain 2, outbred Hartley, and outbred euthymic hairless guinea pigs 2–5 dpi following subcutaneous inoculation using guinea pig-adapted VZV [48]. Recent studies indicate a role for the guinea pig model in increasing our understanding of VZV’s involvement in bowel morbidities. The presence of VZV DNA in enteric neurons following intradermal inoculation of guinea pigs further supports clinical observations that VZV may be linked to bowel-related morbidities, such as colonic pseudoobstruction [51], ulcerative colitis [52], and gastric ulcers [53]. Specifically, VZV DNA has been detected in the ileum and colon using PCR for ORF 40 (putative major capsid protein) and ORF 67 (putative glycoprotein I) following intradermal inoculation of guinea pigs [54,55]. Moreover, in situ hybridization demonstrated the presence of VZV ORF 54 DNA (putatively involved in viral DNA cleavage/packaging [55]) in only myenteric neurons and not in axons, satellite cells, or connective tissue [54]. In summary, VZV studies using guinea pigs have provided experimental support for VZV transmission via aerosolized droplets [49,50], the administration of varicella zoster immunoglobulin (VZIG) as a prophylactic for VZV infection [46], and will potentially extend our understanding of the role of VZV in initiation and/or exacerbation of bowel diseases. However, the lack of studies examining the transcriptional profile during latency in sensory ganglia, the inability to induce VZV reactivation, and the limited availability of immunological tools preclude the wide-spread use of the guinea pig model to study VZV pathogenesis. 

### 2.2. Mice and Rats

VZV infection of mice or rats via subcutaneous, ocular, and intraperitoneal routes results in seroconversion without clinical disease [56,57,58,59]. VZV can reach the ganglia as evidenced by the detection of viral DNA in dorsal root ganglia as early as one month post-subcutaneous or footpad inoculation of rats [56,59]. VZV DNA has been detected in non-neuronal cells, brain, brain stem, liver, kidney, and spleen 1-month post-infection following corneal VZV inoculation of mice, but these findings have not been reproduced [58]. Ganglia harvested 37–42 dpi following intraperitoneal inoculation of two day-old Wistar rats harbored viral DNA as well as transcripts associated with ORF 21 (putative tegument protein [55]) but not ORF 40 [57], a gene expression pattern that has been observed in latently infected human ganglia [60]. In a separate study, ORF 62 and ORF 63 transcripts (putative immediate early proteins 3 and 4, respectively [55]) were detected 18 months following bilateral footpad inoculation of rats [61], similarly to what has been described in latently infected human ganglia [62,63,64]. Unfortunately, the impact of these studies remains to be solidified, as the transcriptional profile during VZV latency in human remains under intense debate. The lack of viral replication, which could be indicative of abortive infection, clinical disease, or reactivation have limited the use of this model to investigate VZV pathogenesis and immunity.

A rat model of VZV-associated pain has been developed in order to gain a better understanding of post herpetic neuralgia (PHN). In this model, Wistar rats are inoculated subcutaneously in the footpad and then subjected to behavioral tests that include measurement of paw withdrawal time in response to mechanical or thermal stimuli [65]. These studies report that the threshold measurements for allodynia and for hyperalgesia of the infected paw decreased significantly 5 dpi, remained low until 35 dpi [65], and resolved by 60 dpi [66]. Footpad inoculation with HSV-1 also results in decreased threshold of pain, but whereas Valacicolvir treatment from day 1–6 post-HSV-1 inoculation abrogated allodynia, treatment 1–10 days post VZV-inoculation did not, suggesting that VZV-induced allodynia may not require active viral replication [66]. Although this model focuses on acute VZV infection rather than reactivation, it highlights unique features of VZV-induced pain and provides a model to investigate novel therapies against PHN. 

### 2.3. SCID-hu Mouse

To circumvent some of the host specificity constraints of VZV, Dr. Arvin and colleagues applied the severe-combined immunodeficient (SCID)-humanized (SCID-hu) mouse model to the study of VZV pathogenesis [67]. In this model, either human fetal thymus and liver tissue are introduced as a conjoint implant (thy/liv) under the kidney capsule of a SCID mouse or human skin is introduced subcutaneously as full-thickness dermal graft [19]. The inoculation of the skin implants in this model with VZV either by scarification or direct injection results in changes characteristic of varicella, notably thickening of the epidermis, the presence of multinucleated cells, and mononuclear cell infiltrate 7 days post-infection (dpi) [19]. This is followed by the appearance of vesicular lesions containing balloon cells with intranuclear inclusions, multinucleated giant cells, and acellular material from degenerated cells enclosed by a keratinized surface layer 14 dpi [19]. 

The infection of thy/liv implants directly inoculated with a suspension of VZV-infected cells revealed that both CD4 and CD8 T cells support VZV replication as evidenced by the presence of both viral DNA and infectious virus [19]. These observations are in line with *in vitro* studies that demonstrated high infectivity of tonsillar T cells by VZV [68]. In addition, intravenous injection of VZV-infected tonsillar T cells, but not VZV-infected fibroblasts, in this SCID-hu model resulted in the formation of varicella characteristic skin lesions in implanted human skin [20], suggesting that T cells play a critical role in trafficking of VZV into the skin.

The SCID-hu mouse model has facilitated a rigorous evaluation of the role of several VZV genes in *vivo*. For example, while previous *in vitro* studies have indicated that ORFs 14, 47, and 66 are dispensable for viral replication (putative glycoprotein C, protein kinase, and protein kinase, respectively [55]) [69,70,71,72], data from studies using the SCID-hu mouse model demonstrate that ORFs 47 and 66 are required for VZV replication in human T cells, and ORFs 47 and 14 are necessary for infection and replication in skin cells [73]. The SCID-hu mouse model has also contributed to our understanding of the innate immune response to VZV in the skin, specifically that blocking IFNα signaling enhances VZV replication at this site [20]. However, the immune deficient nature of the SCID-hu mouse model limits investigating the role of the adaptive immune system in controlling VZV replication and dissemination.

The SCID-hu mouse model has also been adapted to study VZV neurotropism by implanting a human fetal dorsal root ganglia (DRG) under the kidney capsule [74]. The direct inoculation of the DRG with VZV-infected fibroblasts leads to the formation of multinuclear cells, transcription of glycoprotein B, and a robust viral replicative phase [74,75]. Studies using this model have shown that viral DNA is detected 14, 28, and 56 days after direct inoculation of DRG xenografts with VZV [74]. Furthermore, while ORF 31 (putative glycoprotein B [55]), 62, and 63 transcripts were all detected 14 dpi with VZV-S strain, only ORFs 62 and 63 mRNA was detected 56 dpi [74], similar to what has been reported in human ganglia [76,77]; this may be indicative of the establishment of latent infection. A major limitation to this model is that the DRG is inoculated directly rather by natural viremia or transfer from infected skin epithelial cells to nerve endings. 

### 2.4. Nonhuman Primates

Several reports have documented naturally acquired varicella in anthropoid apes [78,79]. In one specific case, a gorilla maintained at the Cincinnati Zoo developed a self-limited vesicular exanthema and generated VZV-specific antibodies during a period of community varicella activity in 1984 [80]. Further analysis revealed the presence of a virus that is “indistinguishable from VZV by restriction enzyme analysis” [80]. These observations prompted inoculation experiments in chimpanzees. However, chimpanzees inoculated subcutaneously with VZV developed viremia, but the papular rash was limited to the site of inoculation [81]. 

There were also several attempts to experimentally inoculate monkeys with VZV, but none resulted in clinical disease. In general, monkeys can be categorized as New World (*Platyrrhini*, e.g., marmosets, spider monkeys) or Old World (*Catarrhines*, e.g., macaques) [82]. New World monkeys inhabit Central and South America, and Old World monkeys are native to Africa, India, and Southeast Asia. The New World monkeys are more phylogenetically distant from *Homo sapiens,* and it is estimated that New World monkeys diverged over 35 million years ago whereas Old World monkeys diverged 10–12 million years ago [83]. VZV inoculation of marmosets (*Callithrix jacchus*) via combined oral-nasal-conjunctival route resulted in seroconversion, no varicella, and an inability to detected viral replication [84]. Similarly, intratracheal VZV inoculation of patas monkeys (*Erythrocebus patas*) leads to seroconversion in the absence of clinical disease or viral replication [85]. Both of these studies are suggestive of abortive infection. Finally, cynomolgus monkeys inoculated intratracheally generated T cell and antibody responses to VZV [86]. VZV DNA was detected in bronchial alveolar lavage (BAL) samples only 4 dpi, which most likely reflects input virus and not replication. Finally, no rash was observed and the establishment of latency was not verified [86]. These data suggest that successful VZV replication only occurs in higher order apes, and that inoculation of both Old and New World monkeys presents similar limitations as those encountered with immune competent rodents.

## 3. Simian Varicella Virus

Records indicate the first outbreak of a varicella-like disease in nonhuman primates in 1967 [87]. Since then, the causative agent has been classified as simian varicella virus (SVV), a primate alphaherpesvirus that is genetically similar to VZV [88,89,90,91]. The SVV and VZV genomes differ slightly in size (SVV: 124,138 bp; VZV: 124,884 bp) and in G+C% content (SVV: 40.4%; VZV: 46%) [6,92]. SVV and VZV genomes share 70–75% DNA similarity [93] and are co-linear with respect to genome organization [90]. Specifically, the similarity between corresponding SVV and VZV genes ranges from 27% (ORF 1, putative membrane protein) to 75% (ORF 31) based on amino acid identity [55]. The only significant variation between the SVV and VZV genomes occurs in the left terminus, where SVV lacks a VZV ORF 2 homologue (putative membrane phosphoprotein [94]) and the SVV genome induces ORF A (truncated homologue of ORF 4 [55]) that is absent from the VZV genome [93,95]. Another difference is the presence of a latency-associated transcript (LAT) that is antisense to ORF 61 (putative immediate early protein 1 [55] in SVV [96,97]. No such transcript has been described for VZV. Numerous studies have confirmed cross-reactive epitopes between SVV and VZV [88,98,99,100]. Given the antigenic, genetic, and clinical similarity of SVV and VZV, SVV infection of nonhuman primates offers an attractive alternative with which to investigate VZV pathogenesis.

### 3.1. Patas Monkeys (*Erythrocebus patas*)

The patas monkey (*Erythrocebus patas*) is a ground-dwelling species of Old World monkey that inhabits open grasslands of Central Africa. It is the only member of the *Erythrocebus* genus, but recent phylogenetic evidence indicates that it is the closest relative of the vervet monkey (*Chlorocebus aethiops*). Historical records report that natural infection of patas monkeys with SVV results in severe, disseminated disease with a 50% fatality rate [101,102]. Therefore, SVV infection of patas monkeys provided an opportunity to evaluate the effectiveness of several antiviral therapeutics, in the prevention of life-threatening complications associated with VZV infection of neonates and immune-compromised patients [103]. Specifically, the administration of phosphonoacetic acetic acid (PAA) intramuscularly 40 h after intratracheal inoculation prevented the development of clinical disease and viral replication in peripheral lymphocytes or in throat swabs [104]. Similar to PAA studies, intravenous administration of acyclovir to monkeys daily for five days resulted in the prevention of varicella, but had no effects on viremia [105]. However, unlike PAA [104], the administration of acyclovir in the absence of infection showed no toxicity [105]. In contrast, adenine arabinoside 5’-monophosphate (ara-AMP) was ineffective against SVV [106]. Patas monkeys have also been used to further define the antigenic relationship between VZV and SVV via challenge experiments [85]. Data from these studies show that intratracheal/subcutaneous inoculation with a clinical VZV strain (CaQu) resulted in the generation of IgG and neutralizing antibodies that cross-reacted against SVV. More importantly, the animals were protected from SVV challenge (intratracheal/subcutaneous inoculation) 35 days later, confirming the antigenic relatedness between the two viruses [85]. 

### 3.2. Cynomolgus Monkeys (*Macaca fascicularis*)

The cynomolgus monkey (*Macaca fascicularis*) is native to Southeast Asia. There are 10 subspecies of *Macaca fascicularis*, and the one most often used in research is commonly referred to as the crab-eating macaque. Initial outbreaks of SVV at the Washington Primate Center between 1969 and 1971 resulted in a total of four deaths and 50 diseased animals out of 184 cynomolgus monkeys at risk [98,107]. In 1989, an outbreak in Japan resulted in 111 clinical cases of SVV and 46 deaths in cynomolgus monkeys [108]. Experimental SVV inoculation of cynomolgus results in the development of vesicular eruption, seroconversion, and virus recovery from urine, bladder, kidney, liver, lymph nodes, and skin biopsies of affected areas 9 and 15 dpi regardless of the route of inoculation (intradermal, intracardiac, intravenous) [109]. Because of the uncertainty surrounding the resolution of acute infection following experimental inoculation, a natural infection model was developed wherein SVV-seronegative monkeys are exposed to animals inoculated intratracheally with 10^4^ PFU SVV. This approach results in the development of varicella rash 10–14 days post-infection and the establishment of latency [110]. To establish a model of herpes zoster, naturally-infected cynomolgus monkeys underwent combined immunosuppression regimen consisting of nonmyeloablative total body irraditation (TBI), tacrolimus, and prednisone [110]. Results from these studies show: (1) detection of SVV DNA in blood days 7, 62, and 105 post-immunosuppression in three of four animals; and (2) the development of zoster rash in one of the four animals [110]. A later study investigated reactivation in animals four months post-SVV infection [111]. Interestingly, they observed the development of zoster rash in three of four cynomolgus macaques 3, 10, and 26 days after starting tacrolimus treatment [111]. Moreover, within two months of immunosuppression-induced reactivation, CD8 T cells were detected in ganglia but their presence did not correlate with ganglionic expression of SVV antigens or SVV ORF 61 anti-sense transcripts [112]. On the other hand, CXCL10 mRNA expression correlated with CD8 T cell infiltration, as previously described in human ganglia collected after VZV reactivation [113]. The major limitation of this natural infection model is the development of lower antibody titers compared to that detected in VZV seropositive humans and an uncharacterized T cell response [110], which could explain the relative ease of reactivation observed in this model (viral DNA detected within seven days).

### 3.3. *Chlorocebus* ssp.

*Chlorocebus* is a genus of medium-sized primates from the Cercopithecidae family of Old World monkeys. There are currently six species defined, including the African green (*C. sabaeus*), grivet (*C. aethiops*), and vervet (*C. pygerythrus*) monkey. SVV outbreaks in *C. aethiops* at the Liverpool School of Tropical Medicine (UK) (1966) resulted in nine deaths out of the 17 animals at risk [107]. An outbreak of SVV at the Delta Regional Primate Research Center (Louisiana) in 1984 resulted in deaths of five of 9 *C. aethiops* that exhibited clinical disease [114]. Severe disseminated disease following subcutaneous and intratracheal infection of *C. aethiops* that is reminiscent of fatal human VZV infection in immunodeficient patients has been reported [115]. Interestingly, intramuscular injections of VZV glycoproteins I, II, and III (gE, gB, and gH, respectively) did not prevent viremia or severe disseminated rash following intratracheal challenge with SVV in *C. aethiops* [116]. Experimental intratracheal inoculation of African green monkeys results in persistent viremia with the detection of SVV DNA in lung, and liver until 10 months post-infection [117]. Moreover, SVV DNA persists in CD4 and CD8 T cells for months to years following intratracheal inoculation of African green monkeys [118]. To address the issues of persistent viremia and severe disease, a similar natural SVV infection model as that described for cynomolgus macaques was adopted [119]. Naturally infected African green monkeys clear acute viremia [119]. In addition, SVV DNA was detected in ganglia from seven of 10 monkeys that did not experience rash, suggesting that successful ganglionic infection may be more dependent on the hematogenous route of viral trafficking than retrograde transport from infected epithelial cells [120]. Studies using naturally infected animals uncovered that similar to other herpesviruses, SVV encodes a latency-associated transcript (LAT) that is antisense to SVV ORF 61 mRNA [96]. Finally, this natural infection model of SVV has been used to investigate SVV reactivation. These studies demonstrate that while one of four latently infected African green monkeys develop zoster rash following tacrolimus treatment, three out of four develop zoster rash when this treatment was supplemented with nonmyeloablative-TBI [111]. However, as mentioned above for cynomolgus macaque natural infection model, a major limitation of these studies is the low antibody titer and uncharacterized T cell response. 

### 3.4. Rhesus Macaque (*Macaca mulata*)

Rhesus macaques (*Macaca mulatta*), also called rhesus monkeys, are Old World monkeys native to northern India, Bangladesh, Pakistan, Nepal, Burma, Thailand, Afghanistan, Vietnam, and southern China. Rhesus macaques have been used in numerous clinical and laboratory investigations, including the evaluation of several vaccines and antivirals. The first documentation of a mild exanthematous disease in rhesus macaques was in May 1969 at the Washington Regional Primate Research Center (Seattle, WA) [98]. Nineteen rhesus macaques exhibited clinical signs of SVV infection but in stark contrast to other macaque species, no casualties were noted [98]. Experimental SVV inoculation of young Indian rhesus macaques via the intrabronchial route reproduces the hallmarks of acute VZV infection in humans, including: (1) viremia; (2) generalized varicella; (3) T and B cell responses; (3) resolution of viremia and varicella; and (4) establishment of latency in only ganglionic neurons [121]. Intratracheal infection of Chinese rhesus macaques with SVV also results in viremia, the development of T and B cell responses and the establishment of latency only in ganglionic neurons; however, no rash was observed [122]. The lack of clinical symptoms in this study may be due to differences in SVV susceptibility between Indian and Chinese origin macaques. Differences in pathogenesis between these two sub-species have also been observed with SIV infection [123,124]. Differences in the route of inoculation (intrabronchial *versus* intratracheal) could also influence development of disease.

Analysis of viral transcription levels during acute and latent SVV infection in young rhesus macaques shows that all known SVV ORFs are expressed to detectable levels in bronchial alveolar lavage samples [97]. Moreover, a large number of transcripts is also detected in peripheral blood [97]. In contrast, latency is associated with a limited transcriptional profile dominated by SVV ORF 61 transcripts [97]. As described for African green monkeys infected with SVV, latently infected ganglia from rhesus macaques also contain an anti-sense transcript associated with ORF 61, which were ~10x more prevalent than sense transcripts [121]. We have recently investigated the role of ORF 61 on disease severity, development of immune responses, and establishment of latency *in vivo* during SVV infection [125]. Infection of rhesus macaques with a recombinant virus lacking ORF 61 (SVVΔORF61) led to a significant decrease in SVV replication and transcription [125]. Moreover, the absence of ORF 61 resulted in increased infiltration and proliferation of plasmacytoid dendritic cells at the site of infection, as well as an increase in type I interferon gene expression [125]. These data suggest that ORF 61 may be a key regulator in viral gene expression and may interfere with the host anti-viral innate immune response *in vivo*.

An interesting study showed that nonmyeloablative-TBI of a cohort of rhesus macaques resulted in severe disseminated varicella in one of the animals, similar to what is observed with immune compromised persons infected with VZV [126]. Further investigations revealed that a cynomolgus macaque housed in the same room was seropositive for SVV before nonmyeloablative-TBI [126]. Observations of increased SVV-specific IgG titers following nonmyeloablative-TBI in the absence of varicella rash led to the hypothesis that this cynomolgus monkey experienced an episode of subclinical reactivation, which led to the shedding of infectious SVV and in primary infection of the seronegative rhesus macaque [126]. 

Given the clinical, virological, and immunological similarities between SVV infection of rhesus macaques and VZV infection of humans, this animal model will undoubtedly yield critical insights into VZV pathogenesis and host response.

## 4. Conclusions

Our understanding of VZV biology has increased substantially since it was first discovered over a century ago. However, due to the strict host-specificity of infection and cell-associated nature, our knowledge of host-pathogen interaction regarding VZV infection remains incomplete. Numerous efforts have been made to develop adequate animal models of VZV infection, but these models remain limited. Nevertheless, the ability of VZV to establish latency in sensory ganglia of these animals offers an opportunity to identify viral genes that are critical to the establishment and/or maintenance of viral latency. The rhesus macaque model of SVV infection recapitulates key clinical and virological features of VZV infection. Specifically, immune competent rhesus macaques develop a benign disease that closely resembles chickenpox, develop a immune response with similar kinetics to what is observed in VZV infection in children, and acute infection is resolved concurrent with the established latency in sensory ganglia. Therefore, data generated using this model will contribute to our understanding of pathogenesis and host response during VZV infection.

One of the most important aspects in the development of an ideal animal model of VZV infection is reactivation, which has not yet been experimentally induced in the rhesus macaque model. Reactivation has been observed in both cynomolgus and African green monkeys receiving nonmyeloablative-TBI that did not ablate bone marrow and/or treated with immune suppressants [110,111]. Indeed, zoster rash was observed in three of four cynomolgus monkeys 1–2 weeks following tacrolimus treatment, in four of four African green monkeys 1–2 weeks following combined nonmyeloablative-TBI and tacrolimus treatment, but only one of four African green monkeys (18 days after irradiation) following TBI alone [111]. These rates are significantly higher than those in observed in patients receiving immunosuppressive treatments. For instance, following treatment with steroids and calceneurin inhibitors (including tacrolimus), HZ incidence in solid organ transplant recipients (kidney, heart, lung and liver) is between 7.4% (renal) and 16.8% (heart) [127,128]. Moreover, reactivation does not occur until 8–90 months post-transplantation and immune suppression [128,129]. Indeed the rate of reactivation observed in the Mahalingam *et al.*, study above are more in line with those observed in humans receiving bone marrow ablating TBI for autologous bone marrow transplantation where the rates of HZ are 77–84% HZ [130,131]. One potential explanation for the high incidence of SVV reactivation in these studies is the low antibody titers achieved with the “natural” SVV infection model (1:4 neutralizing titers *versus* 1:15800 in experimentally SVV inoculated rhesus macaques and 1:9400 in VZV infected humans) [132,133]. Thus, future work will need to be aimed at developing an experimental reactivation model using the experimentally inoculated rhesus macaque model where the role of T cell *versus* humoral immunity in the prevention of HZ can be evaluated. 

Another area that remains poorly understood is the VZV transcriptional profile during latency. Several studies have investigated VZV transcription in latently infected human sensory ganglia using various methods including quantitative RT-PCR, in-situ hybridization, cDNA libraries and northern blot hybridization. Transcripts associated with VZV ORFs 4, 21, 29, 62, 63, and 66 are detected most often [60,77,134,135,136,137,138]. However, transcripts associated with ORFs 11, 18, 21, 40, 41, 43, 57, 66 and 68 were also reported. One potential explanation for the divergent results and the presence of transcripts from ORFs associated with DNA replication and/or structural genes the delay between death and harvest of ganglia. Moreover, ganglia are often collected from older individuals suffering from a number of co-morbidities such as cancer, which could impact immunological control and thereby viral gene expression. This an area of research that the SVV model could contribute considerably to since tissue harvest and health/immune status of the animal are tightly controlled. Indeed, studies using this model have shown that gene transcription is similar when ganglia are harvested immediately post euthanasia or 30 hrs later [139]. However, these observations need to be extended since ganglia from only two animals and a limited number of ORFs (21, 29, 40, 61, 62, 63, and 66) were examined in this study.

Efforts towards developing an animal model wherein VZV can cause clinical disease should also continue. These efforts will most likely require adapting VZV to growing in cells derived from rhesus macaques including potentially passaging the virus *in vivo*. An animal model that can mimic hallmarks of varicella and zoster using VZV would be of tremendous value to the field and would greatly advance the development of more efficacious second-generation vaccines and antivirals against VZV.

## References

[1] Cohen J., Straus S.E., Arvin A.M. (2007). Varicella-zoster virus replication, pathogenesis, and management.

[2] Puvion-Dutilleul F., Pichard E., Laithier M., Leduc E.H. (1987). Effect of dehydrating agents on DNA organization in herpes viruses. J. Histochem. Cytochem..

[3] Almeida J.D., Howatson A.F., Williams M.G. (1962). Morphology of varicella (chicken pox) virus. Virology.

[4] Cok M.L., Stevens J.G. (1970). Replication of varicella-zoster virus in cell culture: An ultrastructural study. J. Ultrastruct. Res..

[5] Cohen J.I. (2010). The varicella-zoster virus genome. Curr. Top. Microbiol. Immunol..

[6] Davison A.J., Scott J.E. (1986). The complete DNA sequence of varicella-zoster virus. J. Gen. Virol..

[7] Cohen J.I., Brunell P.A., Straus S.E., Krause P.R. (1999). Recent advances in varicella-zoster virus infection. Ann. Intern. Med..

[8] Leclair J.M., Zaia J.A., Levin M.J., Congdon R.G., Goldmann D.A. (1980). Airborne transmission of chickenpox in a hospital. N. Engl. J. Med..

[9] Sawyer M.H., Chamberlin C.J., Wu Y.N., Aintablian N., Wallace M.R. (1994). Detection of varicella-zoster virus DNA in air samples from hospital rooms. J. Infect. Dis..

[10] Suzuki K., Yoshikawa T., Tomitaka A., Matsunaga K., Asano Y. (2004). Detection of aerosolized varicella-zoster virus DNA in patients with localized herpes zoster. J. Infect. Dis..

[11] Grose C. (1981). Variation on a theme by fenner: The pathogenesis of chickenpox. Pediatrics.

[12] Heininger U., Seward J.F. (2006). Varicella. Lancet.

[13] Morrow G., Slobedman B., Cunningham A.L., Abendroth A. (2003). Varicella-zoster virus productively infects mature dendritic cells and alters their immune function. J. Virol..

[14] Abendroth A., Morrow G., Cunningham A.L., Slobedman B. (2001). Varicella-zoster virus infection of human dendritic cells and transmission to T cells: Implications for virus dissemination in the host. J. Virol..

[15] Taylor S.L., Moffat J.F. (2005). Replication of varicella-zoster virus in human skin organ culture. J. Virol..

[16] Huch J.H., Cunningham A.L., Arvin A.M., Nasr N., Santegoets S.J., Slobedman E., Slobedman B., Abendroth A. (2010). Impact of varicella-zoster virus on dendritic cell subsets in human skin during natural infection. J. Virol..

[17] Mohsen A.H., McKendrick M. (2003). Varicella pneumonia in adults. Eur. Respir. J..

[18] Chiner E., Ballester I., Betlloch I., Blanquer J., Aguar M.C., Blanquer R., Fernandez-Fabrellas E., Andreu A.L., Briones M., Sanz F. (2010). Varicella-zoster virus pneumonia in an adult population: Has mortality decreased?. Scand. J. Infect. Dis..

[19] Moffat J.F., Stein M.D., Kaneshima H., Arvin A.M. (1995). Tropism of varicella-zoster virus for human CD4+ and CD8+ t lymphocytes and epidermal cells in SCID-hu mice. J. Virol..

[20] Ku C.-C., Zerboni L., Ito H., Graham B.S., Wallace M., Arvin A.M. (2004). Varicella-zoster virus transfer to skin by T cells and modulation of viral replication by epidermal cell interferon-{alpha}. J. Exp. Med..

[21] Eshleman E., Shahzad A., Cohrs R.J. (2011). Varicella zoster virus latency. Future Virol..

[22] Bearer E.L., Breakefield X.O., Schuback D., Reese T.S., LaVail J.H. (2000). Retrograde axonal transport of herpes simplex virus: Evidence for a single mechanism and a role for tegument. Proc. Natl. Acad. Sci. USA.

[23] Haanpaa M., Laippala P., Nurmikko T. (2000). Allodynia and pinprick hypesthesia in acute herpes zoster, and the development of postherpetic neuralgia. J. Pain Symptom Manage..

[24] Cohrs R.J., Mehta S.K., Schmid D.S., Gilden D.H., Pierson D.L. (2008). Asymptomatic reactivation and shed of infectious varicella zoster virus in astronauts. J. Med. Virol..

[25] Mehta S.K., Cohrs R.J., Forghani B., Zerbe G., Gilden D.H., Pierson D.L. (2004). Stress-induced subclinical reactivation of varicella zoster virus in astronauts. J. Med. Virol..

[26] Amanna I.J., Carlson N.E., Slifka M.K. (2007). Duration of humoral immunity to common viral and vaccine antigens. N. Engl. J. Med..

[27] Insinga R.P., Itzler R.F., Pellissier J.M., Saddier P., Nikas A.A. (2005). The incidence of herpes zoster in a United States administrative database. J. Gen. Intern. Med..

[28] Gershon A.A., Gershon M.D., Breuer J., Levin M.J., Oaklander A.L., Griffiths P.D. (2010). Advances in the understanding of the pathogenesis and epidemiology of herpes zoster. J. Clin. Virol..

[29] Gilden D., Nagel M.A., Mahalingam R., Mueller N.H., Brazeau E.A., Pugazhenthi S., Cohrs R.J. (2009). Clinical and molecular aspects of varicella zoster virus infection. Future Neurol..

[30] Helgason S., Petursson G., Gudmundsson S., Sigurdsson J.A. (2000). Prevalence of postherpetic neuralgia after a first episode of herpes zoster: Prospective study with long term follow up. BMJ.

[31] Liesegang T.J. (2008). Herpes zoster ophthalmicus natural history, risk factors, clinical presentation, and morbidity. Ophthalmology.

[32] Yeo S.W., Lee D.H., Jun B.C., Chang K.H., Park Y.S. (2007). Analysis of prognostic factors in Bell's palsy and Ramsay Hunt syndrome. Auris Nasus Larynx.

[33] Gilden D.H., Cohrs R.J., Mahalingam R. (2003). Clinical and molecular pathogenesis of varicella virus infection. Viral Immunol..

[34] Gilden D.H., Wright R.R., Schneck S.A., Gwaltney J.M., Mahalingam R. (1994). Zoster sine herpete, a clinical variant. Ann. Neurol..

[35] Lewis G.W. (1958). Zoster sine herpete. Br. Med. J..

[36] Easton H.G. (1970). Zoster sine herpete causing acute trigeminal neuralgia. Lancet.

[37] Oxman M.N., Levin M.J., Johnson G.R., Schmader K.E., Straus S.E., Gelb L.D., Arbeit R.D., Simberkoff M.S., Gershon A.A., Davis L.E. (2005). A vaccine to prevent herpes zoster and postherpetic neuralgia in older adults. N. Engl. J. Med..

[38] Dworkin M.S., Jennings C.E., Roth-Thomas J., Lang J.E., Stukenberg C., Lumpkin J.R. (2002). An Outbreak of Varicella among children attending preschool and elementary school in Illinois. Clin. Infect. Dis..

[39] Galil K., Lee B., Strine T., Carraher C., Baughman A.L., Eaton M., Montero J., Seward J. (2002). Outbreak of varicella at a day-care center despite vaccination. N. Engl. J. Med..

[40] Vazquez M., LaRussa P.S., Gershon A.A., Niccolai L.M., Muehlenbein C.E., Steinberg S.P., Shapiro E.D. (2004). Effectiveness over time of varicella vaccine. JAMA.

[41] Marin M., Zhang J.X., Seward J.F. (2011). Near elimination of varicella deaths in the US after implementation of the vaccination program. Pediatrics.

[42] Oxman M.N., Levin M.J. (2008). Vaccination against herpes zoster and postherpetic neuralgia. J. Infect. Dis..

[43] Levin M.J., Oxman M.N., Zhang J.H., Johnson G.R., Stanley H., Hayward A.R., Caulfield M.J., Irwin M.R., Smith J.G., Clair J. (2008). Varicella-zoster virus-specific immune responses in elderly recipients of a herpes zoster vaccine. J. Infect. Dis..

[44] Schmader K.E., Oxman M.N., Levin M.J., Johnson G., Zhang J.H., Betts R., Morrison V.A., Gelb L., Guatelli J.C., Harbecke R. (2012). Persistence of the efficacy of zoster vaccine in the shingles prevention study and the short-term persistence substudy. Clin. Infect. Dis..

[45] Kinchington P.R., Goins W.F. (2011). Varicella zoster virus-induced pain and post-herpetic neuralgia in the human host and in rodent animal models. J. Neurovirol..

[46] Myers M.G., Connelly B.L., Stanberry L.R. (1991). Varicella in hairless guinea pigs. J. Infect. Dis..

[47] Myers M.G., Stanberry L.R., Edmond B.J. (1985). Varicella-zoster virus infection of strain 2 guinea pigs. J. Infect. Dis..

[48] Lowry P.W., Sabella C., Koropchak C.M., Watson B.N., Thackray H.M., Abbruzzi G.M., Arvin A.M. (1993). Investigation of the pathogenesis of varicella-zoster virus infection in guinea pigs by using polymerase chain reaction. J. Infect. Dis..

[49] Myers M.G., Duer H.L., Hausler C.K. (1980). Experimental infection of guinea pigs with varicella-zoster virus. J. Infect. Dis..

[50] Matsunaga Y., Yamanishi K., Takahashi M. (1982). Experimental infection and immune response of guinea pigs with varicella-zoster virus. Infect. Immun..

[51] Nomdedeu J.F., Nomdedeu J., Martino R., Bordes R., Portorreal R., Sureda A., Domingo-Albos A., Rutllant M., Soler J. (1995). Ogilvie's syndrome from disseminated varicella-zoster infection and infarcted celiac ganglia. J. Clin. Gastroenterol..

[52] Keene J.K., Lowe D.K., Grosfeld J.L., Fitzgerald J.F., Gonzales-Crussi F. (1978). Disseminated varicella complicating ulcerative colitis. JAMA.

[53] Milligan K.L., Jain A.K., Garrett J.S., Knutsen A.P. (2012). Gastric ulcers due to varicella-zoster reactivation. Pediatrics.

[54] Chen J.J., Gershon A.A., Li Z., Cowles R.A., Gershon M.D. (2011). Varicella zoster virus (VZV) infects and establishes latency in enteric neurons. J. Neurovirol..

[55] Gray W.L. (2004). Simian varicella: A model for human varicella-zoster virus infections. Rev. Med. Virol..

[56] Sadzot-Delvaux C., Merville-Louis M.P., Delree P., Marc P., Piette J., Moonen G., Rentier B. (1990). An in vivo model of varicella-zoster virus latent infection of dorsal root ganglia. J. Neurosci. Res..

[57] Brunell P.A., Ren L.C., Cohen J.I., Straus S.E. (1999). Viral gene expression in rat trigeminal ganglia following neonatal infection with varicella-zoster virus. J. Med. Virol..

[58] Wroblewska Z., Valyi-Nagy T., Otte J., Dillner A., Jackson A., Sole D.P., Fraser N.W. (1993). A mouse model for varicella-zoster virus latency. Microb. Pathog..

[59] Annunziato P., LaRussa P., Lee P., Steinberg S., Lungu O., Gershon A.A., Silverstein S. (1998). Evidence of latent varicella-zoster virus in rat dorsal root ganglia. J. Infect. Dis..

[60] Cohrs R.J., Barbour M.B., Mahalingam R., Wellish M., Gilden D.H. (1995). Varicella-zoster virus (VZV) transcription during latency in human ganglia: Prevalence of VZV gene 21 transcripts in latently infected human ganglia. J. Virol..

[61] Kennedy P.G., Grinfeld E., Bontems S., Sadzot-Delvaux C. (2001). Varicella-zoster virus gene expression in latently infected rat dorsal root ganglia. Virology.

[62] Croen K.D., Ostrove J.M., Dragovic L.J., Straus S.E. (1988). Patterns of gene expression and sites of latency in human nerve ganglia are different for varicella-zoster and herpes simplex viruses. Proc. Natl. Acad. Sci. USA.

[63] Mahalingam R., Wellish M., Wolf W., Dueland A.N., Cohrs R., Vafai A., Gilden D. (1990). Latent varicella-zoster viral DNA in human trigeminal and thoracic ganglia. N. Engl. J. Med..

[64] Meier J.L., Holman R.P., Croen K.D., Smialek J.E., Straus S.E. (1993). Varicella-zoster virus transcription in human trigeminal ganglia. Virology.

[65] Fleetwood-Walker S.M., Quinn J.P., Wallace C., Blackburn-Munro G., Kelly B.G., Fiskerstrand C.E., Nash A.A., Dalziel R.G. (1999). Behavioural changes in the rat following infection with varicella-zoster virus. J. Gen. Virol..

[66] Dalziel R.G., Bingham S., Sutton D., Grant D., Champion J.M., Dennis S.A., Quinn J.P., Bountra C., Mark M.A. (2004). Allodynia in rats infected with varicella zoster virus--a small animal model for post-herpetic neuralgia. Brain Res. Brain Res. Rev..

[67] Ku C.-C., Besser J., Abendroth A., Grose C., Arvin A.M. (2005). Varicella-zoster virus pathogenesis and immunobiology: New concepts emerging from investigations with the SCIDhu mouse model. J. Virol..

[68] Ku C.C., Padilla J.A., Grose C., Butcher E.C., Arvin A.M. (2002). Tropism of varicella-zoster virus for human tonsillar CD4(+) T lymphocytes that express activation, memory, and skin homing markers. J. Virol..

[69] Heineman T.C., Cohen J.I. (1995). The varicella-zoster virus (VZV) open reading frame 47 (ORF47) protein kinase is dispensable for viral replication and is not required for phosphorylation of ORF63 protein, the VZV homolog of herpes simplex virus ICP22. J. Virol..

[70] Heineman T.C., Seidel K., Cohen J.I. (1996). The varicella-zoster virus ORF66 protein induces kinase activity and is dispensable for viral replication. J. Virol..

[71] Kinchington P.R., Ling P., Pensiero M., Gershon A., Hay J., Ruyechan W.T. (1990). A possible role for glycoprotein gpV in the pathogenesis of varicella-zoster virus. Adv. Exp. Med. Biol..

[72] Cohen J.I., Seidel K.E. (1994). Absence of varicella-zoster virus (VZV) glycoprotein V does not alter growth of VZV in vitro or sensitivity to heparin. J. Gen. Virol..

[73] Moffat J.F., Zerboni L., Sommer M.H., Heineman T.C., Cohen J.I., Kaneshima H., Arvin A.M. (1998). The ORF47 and ORF66 putative protein kinases of varicella-zoster virus determine tropism for human T cells and skin in the SCID-hu mouse. Proc. Natl. Acad. Sci. USA.

[74] Zerboni L., Ku C.C., Jones C.D., Zehnder J.L., Arvin A.M. (2005). Varicella-zoster virus infection of human dorsal root ganglia in vivo. Proc. Natl. Acad. Sci. USA.

[75] Reichelt M., Zerboni L., Arvin A.M. (2008). Mechanisms of varicella-zoster virus neuropathogenesis in human dorsal root ganglia. J. Virol..

[76] Mahalingam R., Wellish M., Cohrs R., Debrus S., Piette J., Rentier B., Gilden D.H. (1996). Expression of protein encoded by varicella-zoster virus open reading frame 63 in latently infected human ganglionic neurons. Proc. Natl. Acad. Sci. USA.

[77] Cohrs R.J., Barbour M., Gilden D.H. (1996). Varicella-zoster virus (VZV) transcription during latency in human ganglia: Detection of transcripts mapping to genes 21, 29, 62, and 63 in a cDNA library enriched for VZV RNA. J. Virol..

[78] Heuschele W.P. (1960). Varicella (chicken pox) in three young anthropoid apes. J. Am. Vet. Med. Assoc..

[79] White R.J., Simmons L., Wilson R.B. (1972). Chickenpox in young anthropoid apes: Clinical and laboratory findings. J. Am. Vet. Med. Assoc..

[80] Myers M.G., Kramer L.W., Stanberry L.R. (1987). Varicella in a gorilla. J. Med. Virol..

[81] Cohen J.I., Moskal T., Shapiro M., Purcell R.H. (1996). Varicella in chimpanzees. J. Med. Virol..

[82] Schneider H., Schneider M.P., Sampaio I., Harada M.L., Stanhope M., Czelusniak J., Goodman M. (1993). Molecular phylogeny of the New World monkeys (platyrrhini, primates). Mol. Phylogenet. Evol..

[83] Schrago C.G., Russo C.A. (2003). Timing the origin of New World monkeys. Mol. Biol. Evol..

[84] Provost P.J., Keller P.M., Banker F.S., Keech B.J., Klein H.J., Lowe R.S., Morton D.H., Phelps A.H., McAleer W.J., Ellis R.W. (1987). Successful infection of the common marmoset (callithrix jacchus) with human varicella-zoster virus. J. Virol..

[85] Felsenfeld A.D., Schmidt N.J. (1979). Varicella-zoster virus immunizes patas monkeys against simian varicella-like disease. J. Gen. Virol..

[86] Willer D.O., Ambagala A.P., Pilon R., Chan J.K., Fournier J., Brooks J., Sandstrom P., Macdonald K.S. (2012). Experimental infection of cynomolgus macaques (macaca fascicularis) with human varicella-zoster virus. J. Virol..

[87] Clarkson M.J., Thorpe E., McCarthy K. (1967). A virus disease of captive vervet monkeys (cercopithecus aethiops) caused by a new herpesvirus. Arch. Gesamte. Virusforsch..

[88] Fletcher T.M., Gray W.L. (1992). Simian varicella virus: Characterization of virion and infected cell polypeptides and the antigenic cross-reactivity with varicella-zoster virus. J. Gen. Virol..

[89] Gray W.L., Pumphrey C.Y., Ruyechan W.T., Fletcher T.M. (1992). The simian varicella virus and varicella zoster virus genomes are similar in size and structure. Virology.

[90] Pumphrey C.Y., Gray W.L. (1992). The genomes of simian varicella virus and varicella zoster virus are colinear. Virus Res..

[91] Gray W.L., Starnes B., White M.W., Mahalingam R. (2001). The DNA sequence of the simian varicella virus genome. Virology.

[92] Clarke P., Rabkin S.D., Inman M.V., Mahalingam R., Cohrs R., Wellish M., Gilden D.H. (1992). Molecular analysis of simian varicella virus DNA. Virology.

[93] Gray W.L., Oakes J.E. (1984). Simian varicella virus DNA shares homology with human varicella-zoster virus DNA. Virology.

[94] Sato H., Pesnicak L., Cohen J.I. (2002). Varicella-zoster virus open reading frame 2 encodes a membrane phosphoprotein that is dispensable for viral replication and for establishment of latency. J. Virol..

[95] Gray W.L. (2010). Simian varicella virus: Molecular virology. Curr. Top. Microbiol. Immunol..

[96] Ou Y., Davis K.A., Traina-Dorge V., Gray W.L. (2007). Simian varicella virus expresses a latency-associated transcript that is antisense to open reading frame 61 (ICP0) mRNA in neural ganglia of latently infected monkeys. J. Virol..

[97] Meyer C., Kerns A., Barron A., Kreklywich C., Streblow D.N., Messaoudi I. (2011). Simian varicella virus gene expression during acute and latent infection of rhesus macaques. J. Neurovirol..

[98] Blakely G.A., Lourie B., Morton W.G., Evans H.H., Kaufmann A.F. (1973). A varicella-like disease in macaque monkeys. J. Infect. Dis..

[99] Felsenfeld A.D., Schmidt N.J. (1977). Antigenic relationships among several simian varicella-like viruses and varicella-zoster virus. Infect. Immun..

[100] Felsenfeld A.D., Schmidt N.J. (1975). Immunological relationship between delta herpesvirus of patas monkeys and varicells-zoster virus of humans. Infect. Immun..

[101] Wolf R.H., Smetana H.F., Allen W.P., Felsenfeld A.D. (1974). Pathology and clinical history of delta herpesvirus infection in patas monkeys. Lab. Anim. Sci..

[102] Allen W.P., Felsenfeld A.D., Wolf R.H., Smetana H.F. (1974). Recent studies on the isolation and characterization of delta herpesvirus. Lab. Anim. Sci..

[103] Iltis J.P., Arrons M.C., Castellano G.A., Madden D.L., Sever J.L., Curfman B.L., London W.T. (1982). Simian varicella virus (delta herpesvirus) infection of patas monkeys leading to pneumonia and encephalitis. Proc. Soc. Exp. Biol. Med..

[104] Felsenfeld A.D., Abee C.R., Gerone P.J., Soike K.F., Williams S.R. (1978). Phosphonoacetic acid in the treatment of simian varicella. Antimicrob. Agents Chemother..

[105] Soike K.F., Felsenfeld A.D., Gerone P.J. (1981). Acyclovir treatment of experimental simian varicella infection of monkeys. Antimicrob. Agents Chemother..

[106] Soike K.F., Felsenfeld A.D., Gibson S., Gerone P.J. (1980). Ineffectiveness of adenine arabinoside and adenine arabinoside 5'-monophosphate in simian varicella infection. Antimicrob. Agents Chemother..

[107] Soike K.F. (1992). Simian varicella virus infection in african and asian monkeys. The potential for development of antivirals for animal diseases. Ann. N. Y. Acad. Sci..

[108] Gray W.L. (2008). Simian varicella in old world monkeys. Comp. Med..

[109] Wenner H.A., Barrick S., Abel D., Seshumurty P. (1975). The pathogenesis of simian varicella virus in cynomolgus monkeys. Proc. Soc. Exp. Biol. Med..

[110] Mahalingam R., Traina-Dorge V., Wellish M., Lorino R., Sanford R., Ribka E.P., Alleman S.J., Brazeau E., Gilden D.H. (2007). Simian varicella virus reactivation in cynomolgus monkeys. Virology.

[111] Mahalingam R., Traina-Dorge V., Wellish M., Deharo E., Singletary M.L., Ribka E.P., Sanford R., Gilden D. (2010). Latent simian varicella virus reactivates in monkeys treated with tacrolimus with or without exposure to irradiation. J. Neurovirol..

[112] Ouwendijk W.J., Abendroth A., Traina-Dorge V., Getu S., Steain M., Wellish M., Andeweg A.C., Osterhaus A.D., Gilden D., Verjans G.M. (2013). T-cell infiltration correlates with CXCL10 expression in ganglia of cynomolgus macaques with reactivated simian varicella virus. J. Virol..

[113] Steain M., Gowrishankar K., Rodriguez M., Slobedman B., Abendroth A. (2011). Upregulation of CXCL10 in human dorsal root ganglia during experimental and natural varicella-zoster virus infection. J. Virol..

[114] Lehner N.D., Bullock B.C., Jones N.D. (1984). Simian varicella infection in the African green monkey (Cercopithecus aethiops). Lab. Anim. Sci..

[115] Roberts E.D., Baskin G.B., Soike K., Gibson S.V. (1984). Pathologic changes of experimental simian varicella (Delta herpesvirus) infection in African green monkeys (Cercopithecus aethiops). Am. J. Vet. Res..

[116] Soike K.F., Keller P.M., Ellis R.W. (1987). Immunization of monkeys with varicella-zoster virus glycoprotein antigens and their response to challenge with simian varicella virus. J. Med. Virol..

[117] White T.M., Mahalingam R., Traina-Dorge V., Gilden D.H. (2002). Simian varicella virus DNA is present and transcribed months after experimental infection of adult African green monkeys. J. Neurovirol..

[118] White T.M., Mahalingam R., Traina-Dorge V., Gilden D.H. (2002). Persistence of simian varicella virus DNA in CD4(+) and CD8(+) blood mononuclear cells for years after intratracheal inoculation of african green monkeys. Virology.

[119] Mahalingam R., Traina-Dorge V., Wellish M., Smith J., Gilden D.H. (2002). Naturally acquired simian varicella virus infection in African green monkeys. J. Virol..

[120] Mahalingam R., Wellish M., Soike K., White T., Kleinschmidt-DeMasters B.K., Gilden D.H. (2001). Simian varicella virus infects ganglia before rash in experimentally infected monkeys. Virology.

[121] Messaoudi I., Barron A., Wellish M., Engelmann F., Legasse A., Planer S., Gilden D., Nikolich-Zugich J., Mahalingam R. (2009). Simian varicella virus infection of rhesus macaques recapitulates essential features of varicella zoster virus infection in humans. PLoS Pathog..

[122] Ouwendijk W.J., Mahalingam R., Traina-Dorge V., van Amerongen G., Wellish M., Osterhaus A.D., Gilden D., Verjans G.M. (2012). Simian varicella virus infection of Chinese rhesus macaques produces ganglionic infection in the absence of rash. J. Neurovirol..

[123] Ling B., Veazey R.S., Luckay A., Penedo C., Xu K., Lifson J.D., Marx P.A. (2002). SIV(mac) pathogenesis in rhesus macaques of chinese and indian origin compared with primary HIV infections in humans. AIDS.

[124] Trichel A.M., Rajakumar P.A., Murphey-Corb M. (2002). Species-specific variation in SIV disease progression between Chinese and Indian subspecies of rhesus macaque. J. Med. Primatol..

[125] Meyer C., Kerns A., Haberthur K., Dewane J., Walker J., Gray W., Messaoudi I. (2013). Attenuation of the adaptive immune response in rhesus macaques infected with simian varicella virus lacking open reading frame 61. J. Virol..

[126] Kolappaswamy K., Mahalingam R., Traina-Dorge V., Shipley S.T., Gilden D.H., Kleinschmidt-Demasters B.K., McLeod C.G., Hungerford L.L., DeTolla L.J. (2007). Disseminated simian varicella virus infection in an irradiated rhesus macaque (Macaca mulatta). J. Virol..

[127] Gourishankar S., McDermid J.C., Jhangri G.S., Preiksaitis J.K. (2004). Herpes zoster infection following solid organ transplantation: Incidence, risk factors and outcomes in the current immunosuppressive era. Am. J. Transplant..

[128] Herrero J.I., Quiroga J., Sangro B., Pardo F., Rotellar F., Alvarez-Cienfuegos J., Prieto J. (2004). Herpes zoster after liver transplantation: Incidence, risk factors, and complications. Liver. Transpl..

[129] Fuks L., Shitrit D., Fox B.D., Amital A., Raviv Y., Bakal I., Kramer M.R. (2009). Herpes zoster after lung transplantation: Incidence, timing, and outcome. Ann. Thorac. Surg..

[130] Schuchter L.M., Wingard J.R., Piantadosi S., Burns W.H., Santos G.W., Saral R. (1989). Herpes zoster infection after autologous bone marrow transplantation. Blood.

[131] Locksley R.M., Flournoy N., Sullivan K.M., Meyers J.D. (1985). Infection with varicella-zoster virus after marrow transplantation. J. Infect. Dis..

[132] Dahl H., Marcoccia J., Linde A. (1997). Antigen detection: The method of choice in comparison with virus isolation and serology for laboratory diagnosis of herpes zoster in human immunodeficiency virus-infected patients. J. Clin. Microbiol..

[133] Haberthur K., Engelmann F., Park B., Barron A., Legasse A., Dewane J., Fischer M., Kerns A., Brown M., Messaoudi I. (2011). CD4 T cell immunity is critical for the control of simian varicella virus infection in a nonhuman primate model of VZV infection. PLoS Pathog..

[134] Cohrs R.J., Hurley M.P., Gilden D.H. (2003). Array analysis of viral gene transcription during lytic infection of cells in tissue culture with varicella-zoster virus. J. Virol..

[135] Cohrs R.J., Gilden D.H. (2007). Prevalence and abundance of latently transcribed varicella-zoster virus genes in human ganglia. J. Virol..

[136] Cohrs R.J., Randall J., Smith J., Gilden D.H., Dabrowski C., van Der Keyl H., Tal-Singer R. (2000). Analysis of individual human trigeminal ganglia for latent herpes simplex virus type 1 and varicella-zoster virus nucleic acids using real-time PCR. J. Virol..

[137] Kennedy P.G., Grinfeld E., Bell J.E. (2000). Varicella-zoster virus gene expression in latently infected and explanted human ganglia. J. Virol..

[138] Nagel M.A., Choe A., Traktinskiy I., Cordery-Cotter R., Gilden D., Cohrs R.J. (2011). Varicella-zoster virus transcriptome in latently infected human ganglia. J. Virol..

[139] Mahalingam R., Traina-Dorge V., Wellish M., Deharo E., Golive A., Messaoudi I., Gilden D. (2010). Effect of time delay after necropsy on analysis of simian varicella-zoster virus expression in latently infected ganglia of rhesus macaques. J. Virol..

